# The N-Terminal Region of the BcWCL1 Photoreceptor Is Necessary for Self-Dimerization and Transcriptional Activation upon Light Stimulation in Yeast

**DOI:** 10.3390/ijms241511874

**Published:** 2023-07-25

**Authors:** Matías Guerrero, Carlos Ruiz, Andrés Romero, Luka Robeson, Diego Ruiz, Francisco Salinas

**Affiliations:** 1 Laboratorio de Genómica Funcional, Instituto de Bioquímica y Microbiología, Facultad de Ciencias, Universidad Austral de Chile, Valdivia 5090000, Chile; matias.guerreroc@usach.cl (M.G.); carlos.ruiz02@alumnos.uach.cl (C.R.); aab.romero.q@gmail.com (A.R.); luka.robeson@ug.uchile.cl (L.R.); diego.ruiz@alumnos.uach.cl (D.R.); 2 ANID–Millennium Science Initiative–Millennium Institute for Integrative Biology (iBIO), Santiago 8330025, Chile

**Keywords:** yeast, *Botrytis cinerea*, photoreceptor, blue light, transcriptional activation

## Abstract

The BcWCL1 protein is a blue-light photoreceptor from the fungus *Botrytis cinerea*. This protein has a central role in *B. cinerea* circadian regulation and is an ortholog to WC-1 from *Neurospora crassa*. The BcWCL1 and WC-1 proteins have similar protein domains, including a LOV (Light Oxygen Voltage) domain for light sensing, two PAS (Per Arnt Sim) domains for protein–protein interaction, and a DNA binding domain from the GATA family. Recently, the blue-light response of BcWCL1 was demonstrated in a version without PAS domains (BcWCL1^PAS∆^). Here, we demonstrated that BcWCL1^PAS∆^ is capable of self-dimerization through its N-terminal region upon blue-light stimulation. Interestingly, we observed that BcWCL1^PAS∆^ enables transcriptional activation as a single component in yeast. By using chimeric transcription factors and the luciferase reporter gene, we assessed the transcriptional activity of different fragments of the N-terminal and C-terminal regions of BcWCL1^PAS∆^, identifying a functional transcriptional activation domain (AD) in the N-terminal region that belongs to the 9aaTAD family. Finally, we determined that the transcriptional activation levels of BcWCL1^PAS∆^ AD are comparable to those obtained with commonly used ADs in eukaryotic cells (Gal4 and p65). In conclusion, the BcWCL1^PAS∆^ protein self-dimerized and activated transcription in a blue-light-dependent fashion, opening future applications of this photoreceptor in yeast optogenetics.

## 1. Introduction

*Botrytis cinerea* is one of the most common phytopathogens in the world [1,2]. This fungus responds to light stimulation, promoting conidiation and inhibition of sclerotia formation [3,4,5,6,7]. The *B. cinerea* capacity to respond the light cues is based on photoreceptors, which are specialized proteins capable of light-sensing [8]. Interestingly, the *B. cinerea* genome encodes 11 photoreceptors that potentially can sense light of different wavelengths [9,10,11]. The set of *B. cinerea* photoreceptors includes: two cryptochromes (BcCRY1 and BcCRY2) for UV light detection, four proteins containing LOV (Light Oxygen Voltage) domains (BcWCL1, BcVVD, BcLOV3, and BcLOV4) and responding to blue light, two rhodopsin-like proteins (BOP1 and BOP2) for green light perception, and three phytochromes (BcPHY1, BcPHY2, and BcPHY3) for red light sensing [10]. Among these photoreceptors, LOV-containing photoreceptors are very interesting, since they use FMN or FAD molecules as a cofactor, which are abundant molecules in eukaryotic cells [12,13]. In general, flavin cofactors generate a cysteinyl flavin C4a adduct upon blue light stimulation, generating a conformational change in the LOV domain [14]. These features enable the functional characterization of LOV-based photoreceptors using different biological platforms. For instance, the BcLOV4 photoreceptor has been characterized using two different eukaryotic models: a human cell line (HEK293T) and the budding yeast *Saccharomyces cerevisiae*, both demonstrating that BcLOV4 binds plasma membrane anionic phospholipids in response to blue light [15]. Therefore, *B. cinerea* photobiology is an interesting source of photoreceptors with different biological functions, although their molecular characterization has been only partially addressed.

The BcWCL1 protein is a blue light photoreceptor and transcription factor from the GATA-type Zinc family [10,16,17]. Importantly, the BcWCL1 protein is an ortholog to the WC-1 protein from *Neurospora crassa* (hereafter referred to as NcWC-1), a well-characterized blue light photoreceptor containing a LOV domain [18,19,20]. Furthermore, NcWC-1 is also a transcription factor from the GATA-type Zinc family involved in light response and circadian regulation in *N. crassa* [18,19,20,21]. Besides the LOV domain and DNA binding domain (DBD), the NcWC-1 protein structure also includes two PAS (Per Arnt Sim) domains (PASB and PASC) involved in protein–protein interaction, and two transcriptional activation domains (AD) in the N-terminal and C-terminal regions, respectively [21,22]. Interestingly, the N-terminal region (amino acids 100–200) of NcWC-1 has been validated as a functional AD in *N. crassa* [23]. In the circadian context, NcWC-1 interacts with the WC-2 protein (hereafter referred to as NcWC-2) through its PAS domains, forming the White-Collar Complex (WCC) [21,22]. The WCC binds the *frq* (frequency) promoter, which controls the expression of the negative element in the *N. crassa* circadian clock, the FRQ protein [19]. In *B. cinerea*, BcWCL1 plays a similar role in circadian regulation, also forming the WCC in the nucleus and promoting the light-mediated transcriptional activation of several genes, including *bcfrq* (encoding BcFRQ protein), ortholog to the *frq* gene in *N. crassa* [16,17]. Importantly, the deletion of *bcwcl1* (encoding BcWCL1) and *bcfqr* genes impairs virulence and oxidative stress response in *B. cinerea* [16,24]. Altogether, the importance of BcWCL1 in the *B. cinerea* circadian clock and pathogenesis is propelling new efforts toward its molecular characterization.

Recently, the blue light response of BcWCL1 has been described using the budding yeast *S. cerevisiae* as a biological platform [25]. By using the yeast two-hybrid (Y2H) architecture [26], and replacing the photoreceptor components in a previously described optogenetic system [27] with different versions of BcWCL1 and BcWCL2, we assayed the light-dependent protein–protein interaction between these proteins [25]. As expected, BcWCL1 and BcWCL2 interaction is mainly mediated by the PAS domains with or without light stimulation [25]. Interestingly, in the absence of PAS domains in both proteins (BcWCL1^PAS∆^ and BcWCL2^PAS∆^), the protein–protein interaction becomes light-dependent [25]. Moreover, BcWCL1^PAS∆^ was capable of light-mediated transcriptional activation in the absence of an interacting partner [25]. These results opened important possibilities regarding BcWCL1 photobiology: 1, blue light exerts a conformational change in BcWCL1^PAS∆^ exposing a transcriptional activation domain that is functional in yeast; and 2, BcWCL1^PAS∆^ self-dimerizes upon blue light stimulation.

In this work, we assess the light-dependent transcriptional activity of BcWCL1^PAS∆^ in yeast. By using the Y2H strategy and different versions of BcWCL1^PAS∆^, we demonstrated that BcWCL1^PAS∆^ self-dimerizes through its N-terminal region upon blue light stimulation. Furthermore, we used different fragments of the BcWCL1^PAS∆^ protein to generate chimeric transcription factors, allowing us to identify a functional transcriptional AD in the N-terminal region (amino acids 121–240). Finally, we compared the transcriptional activity of the newly-identified AD with respect to commonly-used ADs in eukaryotic cells (Gal4 and p65), observing that the BcWCL1^PAS∆^-AD can reach higher levels of transcriptional activation in yeast. Altogether, our results support potential applications of the BcWCL1^PAS∆^ photoreceptor in the development of new optogenetic systems in yeast.

## 2. Results

### 2.1. Deletion of PAS Domains in BcWCL1 Does Not Affect the Overall Protein Structure

**Figure 1:**
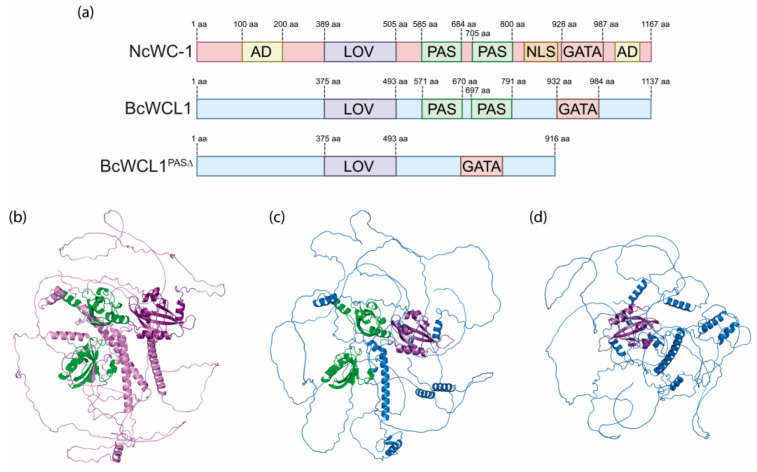
Protein domains and predicted structure of NcWC-1 and BcWCL1. (**a**) Protein domains in NcWC-1, BcWCL1, and BcWCL1^PAS∆^. (**b**) AlphaFold predicted structure of NcWC-1, (**c**) BcWCL1, and (**d**) BcWCL1^PAS∆^, respectively. In all panels, PAS (Per Arnt Sim) domains are shown green, and LOV (Light Oxygen Voltage) domains are shown in purple. Abbreviation: AD, Activation Domain; NLS, Nuclear Localization Sequence; GATA, DNA binding domain from the GATA-type transcription factor family; aa, amino acidic residue.

Previously, we demonstrated that deletion of the PAS domains in BcWCL1 (BcWCL1^PAS∆^) uncover the blue-light response of this protein [25]. In addition, we also observed that BcWCL1^PAS∆^ is capable of transcriptional activation in yeast upon blue-light stimulation [25]. In order to discard a structural effect on BcWCL1 protein due to the deletion of PAS domains, we assess the overall protein structure of BcWCL1 and BcWCL1^PAS∆^ using AlphaFold2 [28]. The results showed that the deletion of PAS domains did not alter the structure of the LOV domain in BcWCL1^PAS∆^ (Figure 1), which is consistent with the light response of this protein. We also extracted from the AlphaFold database [29] the predicted structure for NcWC-1 (Figure 1), *N. crassa* ortholog to BcWCL1, showing a similar globular structure for PAS and LOV domains compared to BcWCL1 (Figure 1B,C). Interestingly, AlphaFold was not able to predict the structure in extensive regions of NcWC-1, BcWCL1, and BcWCL1^PAS∆^ (Figure 1B–D), including the N-terminal and C-terminal regions. Importantly, the N-terminal and C-terminal regions of NcWC-1 have putative transcriptional activation domains (ADs), where the former has been experimentally validated [23]. Thus, the N-terminal and C-terminal regions of NcWCL1 and BcWCL1 proteins could be intrinsically disordered, considering that different ADs such as p53 and NF-κB (p65) have been reported as unfolded [30,31]. In conclusion, a potential AD in the N-terminal region of BcWCL1 (or BcWCL1^PAS∆^) could explain the light-mediated transcriptional activation observed in yeast. Altogether, the deletion of PAS domains in BcWCL1 did not affect the LOV domain structure; however, the predicted structure does not explain the light-dependent transcriptional activation observed in this protein.

### 2.2. BcWCL1^PAS∆^ Self-Dimerizes and Activates Transcription in Response to Light

**Figure 2:**
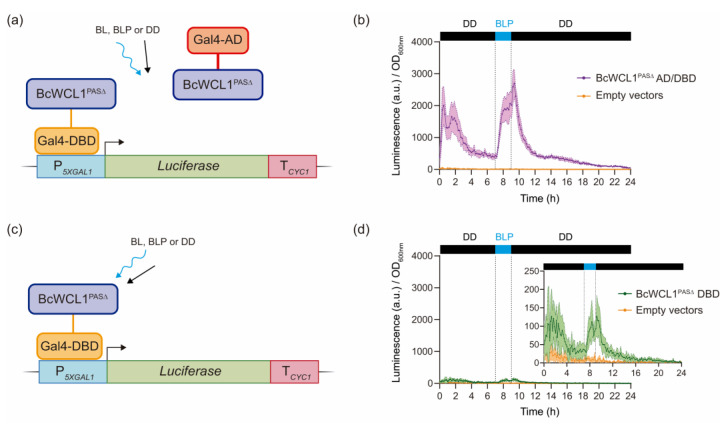
BcWCL1^PAS∆^ protein self-dimerizes and activates transcription upon blue-light stimulation. (**a**) BcWCL1^PAS∆^ protein fused to Gal4-DBD or Gal4-AD was assayed using a yeast two-hybrid architecture and the luciferase reporter. Protein–protein interaction was measured as luciferase expression under three illumination conditions: BL, constant blue light; BLP, a single blue-light pulse of 2 h duration; and DD, constant darkness (full data set in Figures S1 and S2). (**b**) Luciferase expression measured under BLP condition for the protein–protein interaction shown in panel (**a**). (**c**) BcWCL1^PAS∆^ protein fused to Gal4-DBD and assayed as a single component. (**d**) Luciferase expression measured under BLP condition for the single component shown in panel (**c**). In panels (**b**,**d**), luciferase expression is shown as luminescence in arbitrary units (a.u.) divided by the optical density (OD_600nm_) of the yeast cells. The average of six biological replicates with the standard deviation is shown as the color-shaded region.

Initially, we confirmed the BcWCL1^PAS∆^ capacity of light-dependent transcriptional activation in yeast using a Y2H strategy. Hence, we generated a version of BcWCL1^PAS∆^ fused to the Gal4 DNA binding domain (DBD) and AD, BcWCL1^PAS∆^-Gal4-DBD and BcWCL1^PAS∆^-Gal4-AD, respectively. These proteins were assayed for light-mediated protein–protein interaction and transcriptional activation using the luciferase reporter gene (Figure 2A and full data set in Figure S1). The combination of BcWCL1^PAS∆^-Gal4-DBD and BcWCL1^PAS∆^-Gal4-AD results in a strong luciferase expression upon a blue-light pulse (BLP) of 2 h duration (Figure 2B). By contrast, luciferase expression decreased when BcWCL1^PAS∆^-Gal4-DBD was individually assayed (Figure 2C,D), and was absent when BcWCL1^PAS∆^-Gal4-AD was the sole component (Figure S2). Therefore, our results support the supposition that BcWCL1^PAS∆^ is capable of transcriptional activation in yeast, acting as a single component that responds to blue light and, in addition, self-dimerizes upon blue-light stimulation.

**Figure 3:**
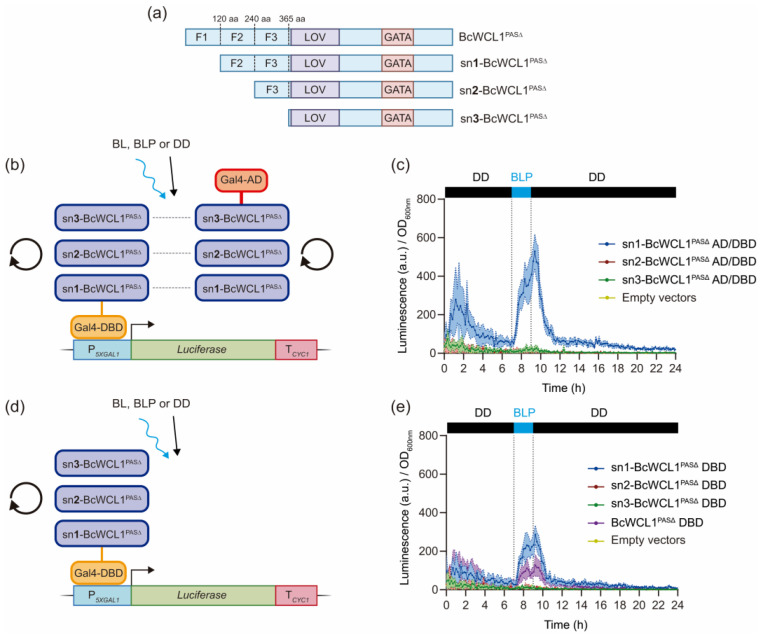
The N-terminal region of BcWCL1^PAS∆^ protein is necessary for self-dimerization and transcriptional activation upon blue-light stimulation. (**a**) The N-terminal region of BcWCL1^PAS∆^ protein was dissected by removing three fragments: amino acids 1–120 (sn1-BcWCL1^PAS∆^), amino acids 121–240 (sn2-BcWCL1^PAS∆^), and amino acids 241–365 (sn3-BcWCL1^PAS∆^). (**b**) Shorter versions of BcWCL1^PAS∆^ were assayed for protein–protein interaction using a yeast two-hybrid architecture and the luciferase reporter. Luciferase expression was assayed under three illumination conditions: BL, constant blue light; BLP, a single blue-light pulse of 2 h duration; and DD, constant darkness (full data set in Figures S3 and S4). (**c**) Luciferase expression measured under BLP condition for the protein–protein interaction shown in panel (**b**). (**d**) BcWCL1^PAS∆^ protein fused to Gal4-DBD and assayed as a single component. (**e**) Luciferase expression measured under BLP condition for the single components shown in panel (**d**). In panels (**c**,**e**), luciferase expression is shown as luminescence in arbitrary units (a.u.) divided by the optical density (OD_600nm_) of the yeast cells. The average of six biological replicates with the standard deviation is shown as the color-shaded region.

We further investigate the molecular mechanism of BcWCL1^PAS∆^ light-dependent self-dimerization in yeast. To this end, we dissected the N-terminal region of this protein to generate a set of shorter versions of BcWCL1^PAS∆^ (Figure 3A). The BcWCL1^PAS∆^ N-terminal region was shortened by removing three sections of similar size: the first 120 amino acids (sn1-BcWCL1^PAS∆^), a further 120 amino acids for a total remotion of 240 amino acids (sn2-BcWCL1^PAS∆^), and finally 365 amino acids corresponding to the entire N-terminal region (sn3- BcWCL1^PAS∆^). These versions were fused to Gal4-DBD and Gal4-AD and then assayed in a Y2H configuration (Figure 3B). The results showed that removing the first 120 amino acids of the N-terminal decreased the light-mediated protein–protein interaction between the sn1-BcWCL1^PAS∆^ versions compared to the full N-terminal protein version (compare luciferase expression in Figure 2B and Figure 3C). Furthermore, the deletion of amino acids 240–365 of the N-terminal region (sn2-BcWCL1^PAS∆^ and sn3-BcWCL1^PAS∆^ versions, respectively) completely abolished the light-mediated protein–protein interaction (Figure 3C and Figure S3). Then, we assayed each shorter version of BcWCL1^PAS∆^ as single components (Figure 3D,E), observing luciferase expression only for sn1-BcWCL1^PAS∆^ upon blue-light stimulation (Figure 3E and full data set in the Figure S4). Interestingly, the removal of the first 120 amino acids (sn1-BcWCL1^PAS∆^) improves the light response of the protein compared to the full N-terminal protein version (Figure 3E). Altogether, amino acids 1–120 of the N-terminal region are important for BcWCL1^PAS∆^ self-dimerization upon blue-light stimulation. In addition, amino acids 121–240 of the N-terminal region are necessary for the light-dependent self-dimerization and transcriptional activation in yeast. Therefore, the results suggest the presence of a functional AD between residues 121 and 240 of the BcWCL1^PAS∆^ N-terminal region.

### 2.3. The Transcriptional Activation Domain of BcWCL1^PAS∆^ Is Localized in the N-Terminal Region between Amino Acids 121–240

**Figure 4:**
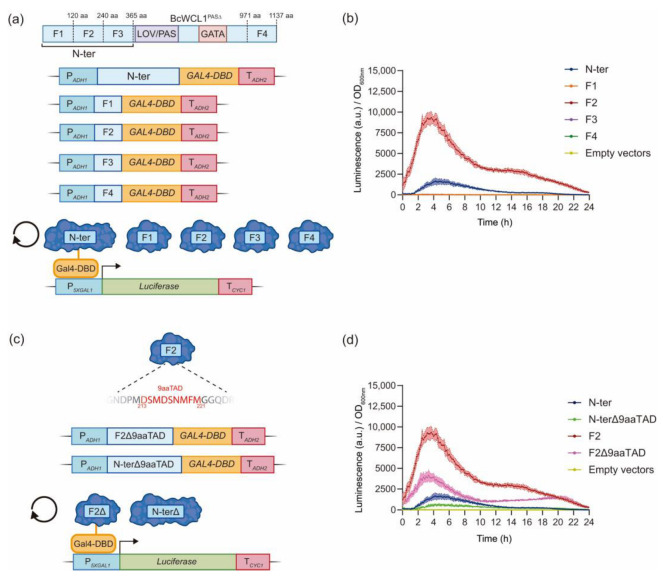
Fragment 2 (F2) inside the N-terminal region of BcWCL1^PAS∆^ is a functional transcriptional activation domain in yeast. (**a**) The full N-terminal region, three fragments of the N-terminal region (F1, F2, and F3), and one fragment of the C-terminal region (F4) of BcWCL1^PAS∆^ were fused to the Gal4-DBD, generating chimeric transcription factors. Transcriptional activity was measured as luciferase expression under constant darkness (shown in this figure) and constant blue-light conditions (full data set in Figures S5 and S7). (**b**) Luciferase expression for the chimeric transcription factors shown in panel (**a**). (**c**) Deletion of the 9aaTAD motif in the chimeric transcription factors carrying F2 and the full N-terminal region of the BcWCL1^PAS∆^ protein. (**d**) Luciferase expression for the chimeric transcription factors shown in panel (**c**). In panels (**b**,**d**), luciferase expression is shown as luminescence in arbitrary units (a.u.) divided by the optical density (OD_600nm_) of the yeast cells. The average of six biological replicates with the standard deviation is shown as the color-shaded region.

In order to demonstrate that the N-terminal region of BcWCL1^PAS∆^ contains a functional AD in yeast, we used a strategy based on the development of chimeric transcription factors [32,33,34,35]. In this strategy, the Gal4-DBD (amino acids 1–149) was fused to the full N-terminal region of BcWCL1^PAS∆^ and three previously delimited fragments of this region: F1 (amino acids 1–120), F2 (amino acids 121–240), and F3 (amino acids 241–365) (Figure 4A). A fourth fragment (F4) from the C-terminal region (amino acids 971–1137) of BcWCL1^PAS∆^ was also assayed (Figure 4A). The functionality of the chimeric transcription factors was tested using the luciferase reporter (Figure 4A). As a result, the full N-terminal region and F2 of BcWCL1^PAS∆^ showed transcriptional activation of the luciferase reporter (Figure 4B), which was independent of blue-light stimulation (Figure S5), consistent with the absence of the LOV domain in the chimeric transcription factors. Interestingly, the F2 showed higher transcriptional activity compared to the full N-terminal region (Figure 4B), suggesting a potential inhibitory effect of F1 over F2 (see discussion section). Therefore, we conclude that BcWCL1^PAS∆^ contains a functional AD between amino acids 121 and 240 of the BcWCL1^PAS∆^ N-terminal region.

Then, we analyzed the N-terminal region of BcWCL1^PAS∆^ for the presence of a 9aaTAD amino acids motif, which is commonly recognized by the eukaryotic transcriptional machinery, and it is present in ADs of transcription factors such as Gal4 and p65 [36]. Interestingly, the analysis revealed the presence of two 9aaTAD motifs in the N-terminal region of BcWCL1^PAS∆^, which are located inside F1 and F2, respectively (Figure S6). As we observed no transcriptional activity for F1 in our previous chimeric transcription factor assays (Figure 4B), we explored the functionality of the 9aaTAD motif inside the transcriptionally active F2. Thus, we generated chimeric transcription factors without the 9aaTAD motif for the F2 and the full N-terminal region of BcWCL1^PAS∆^ (Figure 4C). These chimeric transcription factors were assayed for luciferase expression (Figure 4C), observing a decreased transcriptional activity of the luciferase reporter compared to the wild-type versions (Figure 4D and Figure S7). Interestingly, the reduction observed in F2 suggests that the 9aaTAD motif is important for transcriptional activation, but additional amino acids inside F2 are also necessary for the transcriptional response. In conclusion, the BcWCL1^PAS∆^ contains a functional AD in yeast, which belongs to the 9aaTAD family of ADs, and whose transcriptional activity is between amino acids 121 and 240 of the BcWCL1^PAS∆^ N-terminal region.

### 2.4. Transcriptional Activity of the BcWCL1^PAS∆^ Activation Domain Is Comparable to Other Eukaryotic Activation Domains

**Figure 5:**
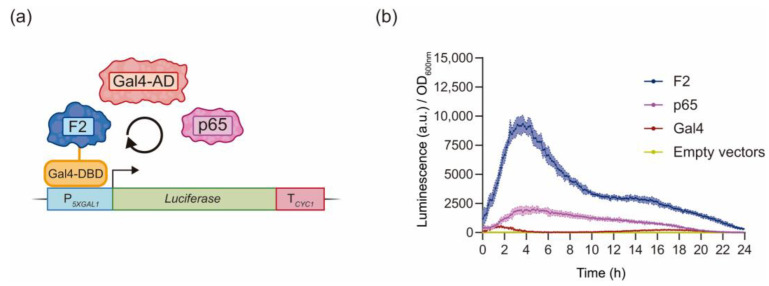
Activation Domain (AD) in fragment 2 (F2) of BcWCL1^PAS∆^ promotes high levels of transcriptional activation in yeast. (**a**) Gal4 and p65 ADs were fused to the Gal4-DBD, generating chimeric transcription factors. Transcriptional activity was measured as luciferase expression under constant darkness condition. (**b**) Luciferase expression for the chimeric transcription factors shown in panel (**a**). Luciferase expression is shown as luminescence in arbitrary units (a.u.) divided by the optical density (OD_600nm_) of the yeast cells. The average of six biological replicates with the standard deviation is shown as the color-shaded region.

Finally, we compared the transcriptional activity of the BcWCL1^PAS∆^ AD (F2) to that of other eukaryotic ADs such as Gal4 and p65. To evaluate this, we developed chimeric transcription factors carrying the Gal4-DBD fused to Gal4 and p65 ADs (Figure 5A). Then, we compared the transcriptional activity of each chimeric transcription factor by measuring the luciferase reporter gene expression (Figure 5A). Interestingly, the chimeric transcription factor with region F2 of BcWCL1^PAS∆^ showed higher levels of luciferase expression compared to Gal4 and p65 ADs (Figure 5B and Figure S8). Furthermore, the results confirm that our approach based on chimeric transcription factors is a viable strategy to assess transcriptional activation in yeast. In conclusion, the BcWCL1^PAS∆^ AD promotes a strong transcriptional activation in yeast, which, combined with its light-mediated self-dimerization, supports future applications of this photoreceptor in the development of novel optogenetic systems.

## 3. Discussion

The blue-light sensing capacity of BcWCL1 was previously demonstrated in a version without PAS domains (BcWCL1^PAS∆^) [25]. Here, we showed that BcWCL1^PAS∆^ self-dimerized upon blue-light stimulation through its N-terminal region (Figure 3). Furthermore, we demonstrate that the N-terminal region of BcWCL1^PAS∆^ contains a functional AD (fragment 2 or F2, amino acids 121–240) in yeast (Figure 4). Interestingly, our experiments suggest that fragment 1 (F1, amino acid 1–120) has an inhibitory effect on the transcriptional activation of F2. This is supported by the results obtained using a shorter version of BcWCL1^PAS∆^ (sn1-BcWCL1^PAS∆^) where F1 was removed, showing an increased light-mediated dimerization and transcriptional activation compared to the BcWCL1^PAS∆^ protein (Figure 3E). Therefore, the inhibitory effect of F1 is light-dependent, where the conformational change (by light) in the LOV domain releases F2 from F1 inhibition and activates transcription. The chimeric transcription factor results also support this conclusion, when F2 was assayed individually (without LOV domain and F1 region), we observed that F2 transcriptional activation is now light-independent and higher than the full N-terminal region (Figure 4 and Figure S5). In general, our findings in BcWCL1^PAS∆^ are in agreement with previously reported results in NcWC-1, where a functional AD (amino acids 100–200) was described in the N-terminal region of this protein [23]. In addition, a putative AD has been described in the C-terminal region of NcWC-1 [21,22], which was not detected in our experiments with the chimeric transcription factor carrying the C-terminal region of BcWCL1^PAS∆^ (Figure 4). Therefore, protein functionality at the N-terminal region of BcWCL1 correlates with its ortholog protein (NcWC-1). 

The chimeric transcription factor strategy used in this work was previously implemented to discover ADs from bacteria [33,35] and in the development of synthetic transcription factors [32,34]. This approach allowed us to dissect the N-terminal region of BcWCL1^PAS∆^, confirming an AD into the F2 that belongs to the 9aaTAD family (Figure 4). Finally, the chimeric transcription factors strategy was validated using the Gal4 and p65 ADs, confirming that BcWCL1^PAS∆^ AD promotes a stronger activation of the luciferase reporter compared to Gal4 and p65 ADs (Figure 5). Therefore, BcWCL1^PAS∆^ is a blue-light photoreceptor that contains a functional AD in yeast, where blue light exerts BcWCL1^PAS∆^ self-dimerization and transcriptional activation through its N-terminal region. 

The BcWCL1^PAS∆^ protein has unique characteristics that promote its application in yeast optogenetics; notably, the presence of a LOV domain sensitive to blue light, self-dimerization upon blue-light stimulation through its N-terminal region, and the presence of an AD in the N-terminal region (amino acids 121–240). Importantly, the deletion of the first 120 amino acids in the N-terminal region improves the light response of the protein as a single component (Figure 3), supporting the application of this shorter version (sn1-BcWCL1^PAS∆^) in yeast optogenetics. Furthermore, luciferase expression activated by BcWCL1^PAS∆^ delf-dimerization is higher under constant BL than in BLP condition (Figures S1–S4), showing a similar behavior compared to the FUN-LOV (FUNgal Light Oxygen Voltage) optogenetic switch [27,37]. In this sense, different single-component optogenetic systems have been developed in yeast, which self-dimerizes and activates transcription upon light illumination [38,39]. The blue-light photoreceptor vivid (VVD) from *N. crassa* contains a LOV domain and self-dimerizes upon blue-light stimulation through its N-terminal region [40]. This photoreceptor was linked to the Gal4 or LexA DBDs, and fused to the Gal4-AD, developing a single-component optogenetic system for light-controlled gene expression that has been implemented in different biological platforms, including yeast [38], mammalian cells [41,42], Zebrafish, and Drosophila [43]. Similarly, the EL222 protein is a blue-light photoreceptor and transcription factor from *Erythrobacter litoralis*, which self-dimerizes upon blue-light stimulation through its LOV domain, binding a target promoter region and activating transcription [44,45,46]. Thus, EL222 has been fused to the VP16 AD and used for light-activated gene expression in different chassis, including yeast [39,47], mammalian cells [48], and Zebrafish [49]. Therefore, single-component optogenetic systems have a wide range of potential applications in different model organisms. 

Altogether, we have demonstrated that the N-terminal region of BcWCL1^PAS∆^ is necessary for self-dimerization and transcriptional activation upon blue-light stimulation. Future experiments should address the possibility of deleting the BcWCL1^PAS∆^ protein region between the LOV domain and the C-terminal end. This could reduce the protein size, favoring the utilization of BcWCL1^PAS∆^ as a single-component optogenetic system for light-controlled gene expression.

## 4. Materials and Methods

### 4.1. Yeast Strains and Culture Conditions

All the experiments were carried out in the BY4741 yeast strain (*MATa*; *his3∆1*; *leu2∆0*; *met15∆0*; *ura3∆0*). This strain was maintained in YPDA (2% glucose, 2% peptone, 1% yeast extract, and 2% agar) at 30 °C. The BY4741 strain was transformed with different plasmids (Section 2.4) and grown in Synthetic Complete (SC) medium (0.67% yeast nitrogen base without amino acids, 2% glucose, 0.2% dropout mix) without the corresponding amino acids for auxotrophic selection. All the yeast strains used and generated in this work are listed in Supplementary Table S1. 

### 4.2. Protein Structure Analysis

Protein domains in NcWC-1 and BcWCL1 were analyzed using the InterPro Scan search tool [50]. In addition, the 3D structure prediction of BcWCL1 and BcWCL1^PAS∆^ was performed using AlphaFold2 [28] under default settings and using the ColabFold server [51]. In the case of NcWC-1, the 3D structure was extracted from the AlphaFold protein structure database (UniProt: Q01371) [29]. Based on these analyses, three fragments of similar size were defined in the N-terminal region of BcWCL1^PAS∆^: F1 (amino acids 1–120), F2 (amino acids 121–240), and F3 (amino acids 241–365). The full N-terminal region of BcWCL1^PAS∆^ was also analyzed (amino acids 1–365). A fourth fragment (F4: amino acids 971–1137) was defined in the C-terminal region of BcWCL1^PAS∆^. All fragments and the full N-terminal region were systematically removed from BcWCL1^PAS∆^ or used to generate chimeric transcription factors (Section 4.3).

The protein sequence of BcWCL1 was scanned for transcriptional ADs carrying the 9aaTAD motif using the online tool described by [52]. This 9aaTAD motif is present in ADs of different eukaryotic transcription factors, including Gal4 (yeast) and p65 (NF-kappa B, animal cells) [52,53]. The prediction of the 9aaTAD motif in BcWCL1 was used to generate chimeric transcription factors without the 9aaTAD motif (Section 4.3).

### 4.3. Genetic Constructs Design and Plasmid Construction

All genetic constructs were designed using the Benchling online platform for molecular biology (https://www.benchling.com/; accessed on 1 March 2022). For light-dependent protein–protein interaction assays, plasmids were constructed based on the components of the FUN-LOV optogenetic switch [27]. For this, pRS423 and pRS425 plasmids were used as backbones for cloning each construct under the *ADH1* promoter control and *ADH2* transcriptional terminator [25,54]. The BcWCL1^PAS∆^ protein was fused to the Gal4-DBD and cloned into the pRS423 plasmid as previously described [25]. In addition, the BcWCL1^PAS∆^ protein was also fused to Gal4-AD and cloned into the pRS425 plasmid. The different versions of BcWCL1^PAS∆^ carrying deletions in the N-terminal (full region or fragments) were fused to Gal4-BDB (amino acids 1–149) or Gal4-AD (amino acids 747–881) and cloned into pRS423 and pRS425 plasmids, respectively.

In the chimeric transcription factor assays, the genetic constructs were generated by fusing the Gal4-DBD (amino acids 1–149) to each N-terminal fragment (F1, F2, and F3), the full N-terminal region, and the C-terminal region (F4). For this, pRS423 plasmid was used as the backbone for cloning each chimeric protein under the *ADH1* promoter control and *ADH2* transcriptional terminator [25,54]. 

In the light-dependent protein–protein interaction and chimeric transcription factor assays, the genetic elements included in the genetic constructs were PCR amplified using Phusion Flash High Fidelity Master mix (Thermo Scientific, Waltham, Massachusetts, USA). The PCR reactions were carried out using primers with 50 bp of overhang between adjacent elements of the genetic construct. This enabled genetic construct assembly using Yeast Recombinational Cloning (YRC) as described by [55]. Briefly, PCR fragments were co-transformed with linearized versions of the pRS423 or pRS425 plasmids using the standard Lithium acetate transformation protocol [56]. Then, YRC-assembled plasmids were extracted from yeast using Zymoprep Yeast Plasmid Miniprep II (Zymo Research, Irvine, California, USA) and used for *E. coli* transformation. Next, YRC-assembled plasmids were confirmed by bacterial colony PCR using GoTaq (Promega, Madison, Wisconsin, USA) under the manufacturer’s instructions. Finally, YRC assembled plasmid were confirmed by sequencing using the Macrogen Sanger sequencing service (Macrogen, Seoul, Republic of Korea). Plasmids used and generated in this work are listed in the Supplementary Table S2. Primers utilized for plasmids assembly are listed in the Supplementary Table S3.

### 4.4. Protein–Protein Interaction and Transcriptional Activity

In protein–protein interaction and chimeric transcription factor assays, the firefly luciferase reporter gene was used to measure transcriptional activity [57]. The luciferase reporter is optimized for in vivo transcriptional measurements in yeast cells, carrying the ARE and PEST sequences for its mRNA and protein degradation, respectively [57]. This destabilized version of the firefly luciferase has 20–30 min of half-life, enabling real-time measurements of transcriptional activity in yeast [57]. The luciferase reporter was controlled by the *5XGAL1* synthetic promoter as previously described [25,27]. These assays were carried out using a Synergy H1M plate reader (Agilent, Santa Clara, California, USA) for simultaneous measurements of luminescence (in arbitrary units; a.u.) and optical density (OD) of the yeast cells. Briefly, yeast cultures were grown overnight in a 96-well plate with 200 µL of SC medium at 30 °C. The next day, a new 96-well plate with optical bottom carrying 285 µL of SC medium and supplemented with 1 mM of luciferin was inoculated with 15 µL of overnight cultures. This plate was incubated in the plate reader for 24 h at 25 °C, allowing the measurements of luminescence (Lum) and OD at 600 nm (OD_600nm_) every 10 min with 30 s of shaking prior to data acquisition [25,54]. 

The protein–protein interaction assays were performed under three illumination conditions: constant darkness (DD), constant blue light (BL), and a single blue-light pulse (BLP) of 2 h duration [25,37,54]. In DD experiments, the plate reader was programmed for continuous kinetics (Gen5 software, Agilent, Santa Clara, California, USA), where the 96-well plate was incubated inside the equipment under DD at 25 °C, measuring Lum and OD_600 mn_ every 10 min. In BL experiments, the plate reader was programmed for discontinuous kinetics (Gen5 software, Agilent, Santa Clara, California, USA), incubating the 96-well plate outside the equipment at room temperature (25 °C) and enabling its illumination by a LED illumination system. This illumination system provides blue light at 466 nm with an intensity of 24 µmol m^2^ s^−1^ [37,54]. Finally, in BLP experiments, the plate reader was programmed for discontinuous kinetics, where the 96-well plate was incubated for 7 h inside the equipment at 25 °C under DD, measuring Lum and OD_600 mn_ every 10 min. Then, the 96-well plate was incubated for 2 h outside of the equipment for blue-light illumination at room temperature (25 °C). After the 2 h illumination pulse, the 96-well plate was incubated inside the equipment at 25 °C for 15 h under DD conditions. In BL and BLP experiments, when incubation was carried out outside the plate reader, the 96 well plate was automatically moved inside the equipment every 10 min for the acquisition of Lum and OD_600nm_. After data acquisition, the 96-well plate was automatically moved outside the plate reader for illumination. The chimeric transcription factor assays were performed under DD and BL conditions, using the same experimental setup (continuous and discontinuous kinetics) described above. All the experiments were performed in six biological replicates during 24 h. 

Data of Lum and OD_600nm_ were normalized by dividing Lum by OD (Lum/OD_600nm_) for each time point. All data sets were analyzed using the GraphPad Prism software version 9.5.1.

## 5. Conclusions

In conclusion, BcWCL1^PAS∆^ protein self-dimerized upon blue-light stimulation through its N-terminal region, where the section between amino acids 121 and 240 (Fragment 2 or F2) is necessary for self-dimerization. As a single component, the BcWCL1^PAS∆^ protein is capable of light-dependent transcriptional activation through the F2 region in yeast. The F2 region contains a functional AD that belongs to the 9aaTAD family, and whose transcriptional activity strength is higher than classical ADs such as Gal4 and p65. Therefore, the BcWCL1^PAS∆^ version of the BcWCL1 photoreceptor is a candidate for the development of a new single-component optogenetic system in yeast.

## Data Availability

Data sets supporting reported results are available upon request to the corresponding author.

## Supplementary Materials

The following supporting information can be downloaded at: https://www.mdpi.com/article/10.3390/ijms241511874/s1.
