# Supplementary material for: The N-Terminal Region of the BcWCL1 Photoreceptor Is Necessary for Self-Dimerization and Transcriptional Activation upon Light Stimulation in Yeast
Source: Int J Mol Sci. 2023 Jul 25;24(15):11874. doi: 10.3390/ijms241511874 (PMC10418492; doi:10.3390/ijms241511874)
Supplement: Supplementary file 1 [file ijms-24-11874-s001.zip › ijms-2505776-supplementary.pdf]

The N-terminal region of the BcWCL1 photoreceptor is necessary for self-dimerization and transcriptional activation upon light stimulation in yeast.

Matías Guerrero [1,2§], Carlos Ruiz [1,2§], Andrés Romero [1,2], Luka Robeson [1], Diego Ruiz [1,2] and Francisco Salinas [1,2*]

1. Laboratorio de Genómica Funcional, Instituto de Bioquímica y Microbiología, Facultad de Ciencias, Universidad Austral de Chile, Valdivia, Chile.

2. ANID–Millennium Science Initiative–Millennium Institute for Integrative Biology (iBIO), Santiago, Chile.

* Correspondence: francisco.salinas@uach.cl (F.S.)

§ These authors contributed equally to this work.

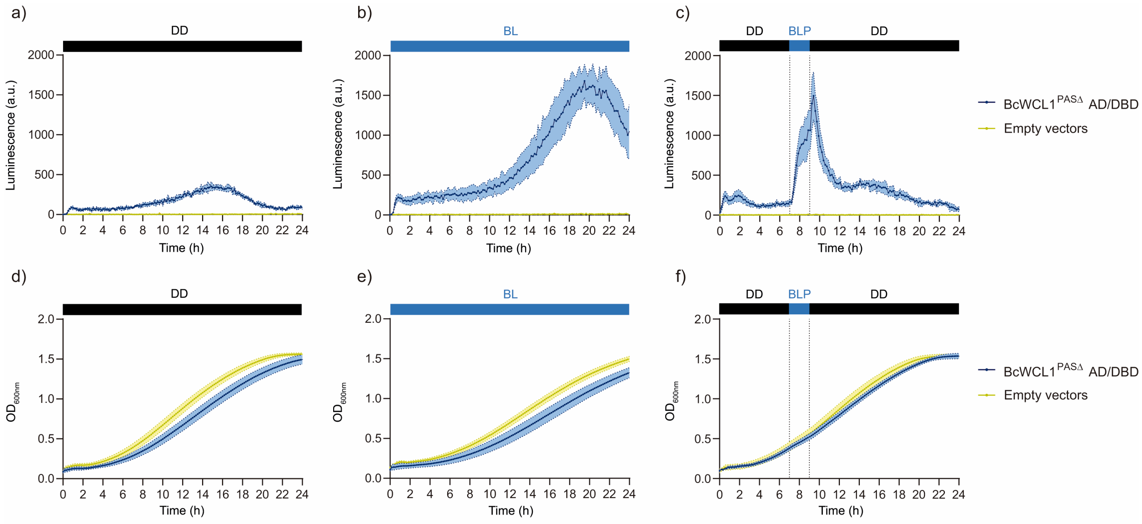

Figure S1: Raw data for BcWCL1$^{PAS\Delta}$ protein-protein interaction. The luciferase expression (panels a, b, and c) measured as luminescence in arbitrary units (a.u.) and the Optical Density (OD) at 600 nm (panels d, e, and f) of the yeast cultures are shown. The protein-protein interaction activates luciferase expression controlled by the *5XGAL1* synthetic promoter under three different experimental conditions: (a and d) constant darkness (DD), (b and e) constant blue-light (BL), and (c and f) a single blue-light pulse (BLP) of 2h (dotted lines). In all panels, the average of six biological replicates with the standard deviation represented as a color shaded region is shown.

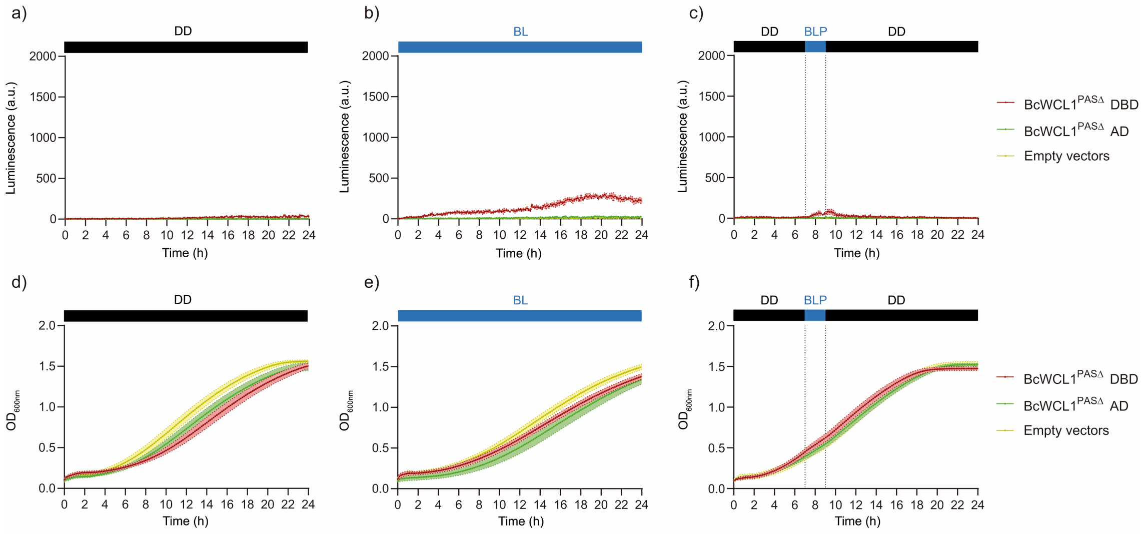

Figure S2: Raw data for BcWCL1$^{PAS\Delta}$ transcriptional activation as single component. The luciferase expression (panels a, b, and c) measured as luminescence in arbitrary units (a.u.) and the Optical Density (OD) at 600 nm (panels d, e, and f) of the yeast cultures are shown. Luciferase expression is controlled by the *5XGAL1* synthetic promoter under three different experimental conditions: (a and d) constant darkness (DD), (b and e) constant blue-light (BL), and (c and f) a single blue-light pulse (BLP) of 2h (dotted lines). In all panels, the average of six biological replicates with the standard deviation represented as a color shaded region is shown.

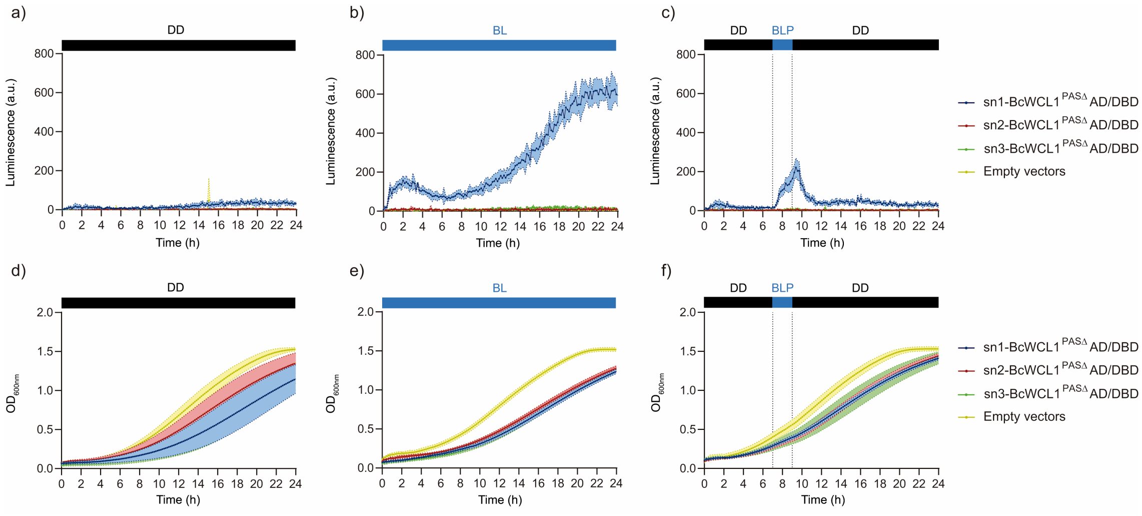

Figure S3: Raw data for protein-protein interaction using shorter versions of BcWCL1$^{PAS\Delta}$. The luciferase expression (panels a, b, and c) measured as luminescence in arbitrary units (a.u.) and the Optical Density (OD) at 600 nm (panels d, e, and f) of the yeast cultures are shown. The protein-protein interaction activates luciferase expression controlled by the *5XGAL1* synthetic promoter under three different experimental conditions: (a and d) constant darkness (DD), (b and e) constant blue-light (BL), and (c and f) a single blue-light pulse (BLP) of 2h (dotted lines). In all panels, the average of six biological replicates with the standard deviation represented as a color shaded region is shown.

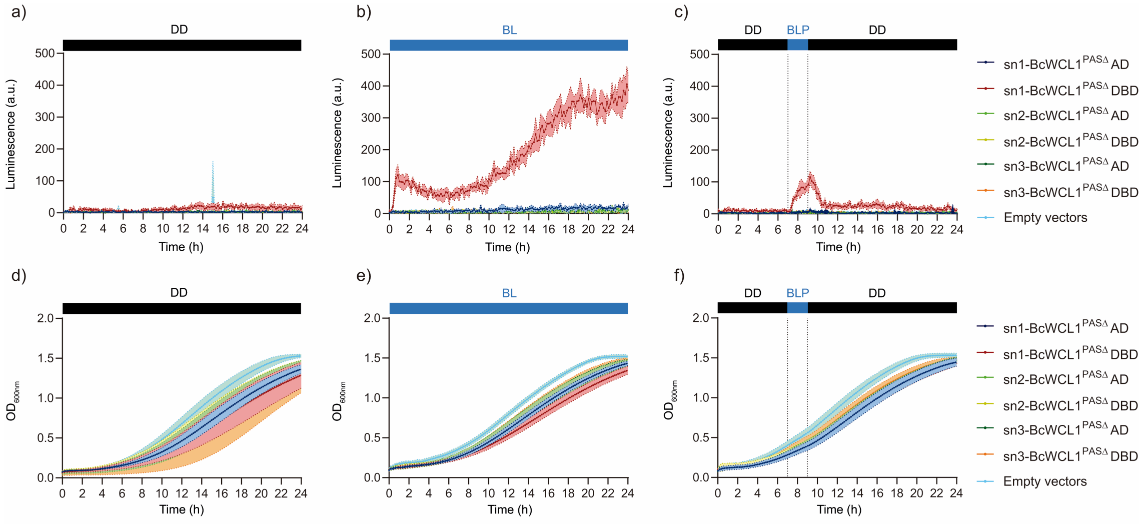

Figure S4: Raw data for transcriptional activation using shorter versions of BcWCL1$^{PAS\Delta}$ as single component. The luciferase expression (panels a, b, and c) measured as luminescence in arbitrary units (a.u.) and the Optical Density (OD) at 600 nm (panels d, e, and f) of the yeast cultures are shown. Luciferase expression is controlled by the *5XGAL1* synthetic promoter under three different experimental conditions: (a and d) constant darkness (DD), (b and e) constant blue-light (BL), and (c and f) a single blue-light pulse (BLP) of 2h (dotted lines). In all panels, the average of six biological replicates with the standard deviation represented as a color shaded region is shown.

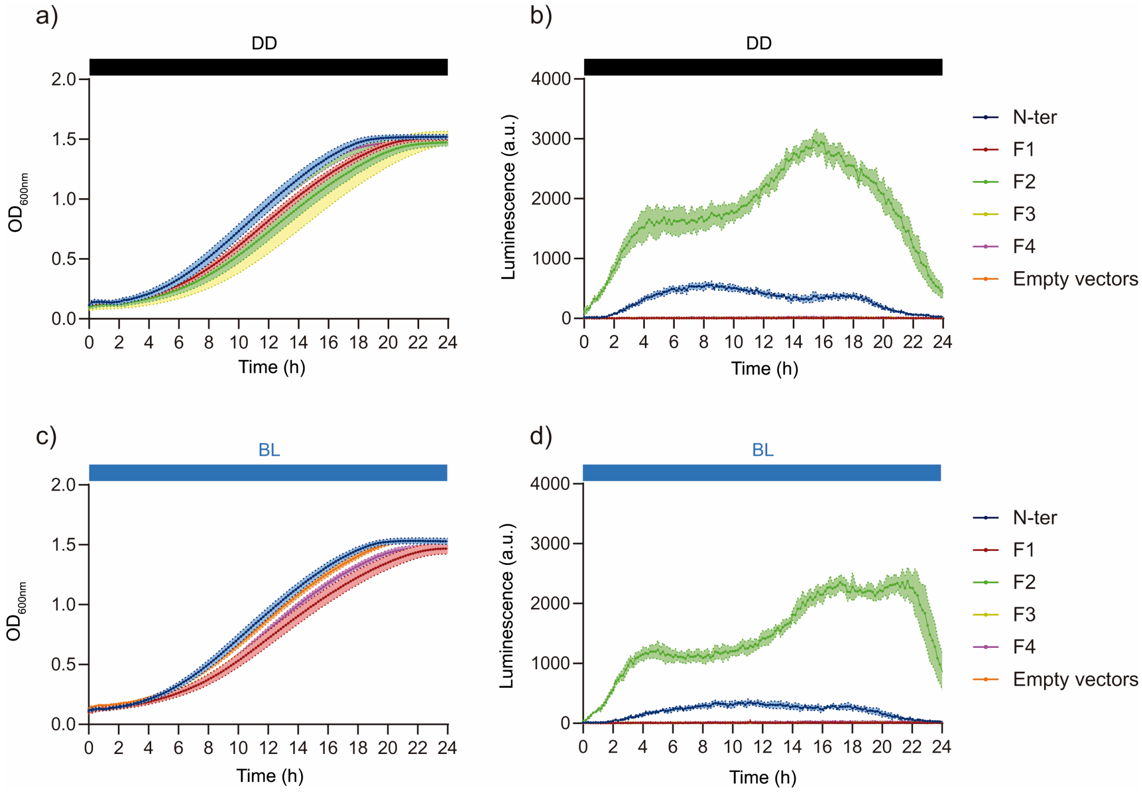

Figure S5: Raw data for chimeric transcription factors carrying different fragments of N-terminal and C-terminal regions of BcWCL1$^{PAS\Delta}$. The Optical Density (OD) at 600 nm (panels a and c) and the luciferase expression (panels b and d) measured as luminescence in arbitrary units (a.u.) of the yeast cultures are shown. OD and luciferase expression were measured under constant darkness (DD, panels a and b) and constant blue light (BL, panels c and d). Luciferase reporter is controlled by the *5XGAL1* synthetic promoter. In all panels, the average of six biological replicates with the standard deviation represented as a color shaded region is shown.

```
MPMTQADLQRQVAQRRAHSRSLSIPQPQPQQQRQQQQPSTQSNGNS
MNMNHNMSGGNSLDEIIMQNNNELQRRQSYSQDFSQDHDRRSSMLEF
GNSDVGLNDFQFGTPTPSSENPMIRRQSLGEMNMNDLYGGGMDSGMG
IMPQMQQLGYSDSVRPNLMDTTMGYGSYSGDMMPGMMSYASIGMDG
MSQDSPMGMYSNDNYSPTVYGNDPMDSMDSNMFMGGQDRGGIRTLG
DENNEEMSSRSSIMGPTAAESLLRTREGMIMRPLEDENNEAMSSRFDVMG
QSPSAMSMPQTLINYNSPSASNAPPNLLTRTSSAAMIPTSVSTPTALTAPDT
SPSGQGITNIYSSTGFDMLGALIRVAKRPN
```

Figure S6: 9aaTAD motifs identified in the N-terminal region of BcWCL1$^{PAS\Delta}$. The N-terminal region of BcWCL1$^{PAS\Delta}$ (residues 1 to 365) was divided in three fragments: F1 (green), F2 (blue), and F3 (purple). The 9aaTAD motifs in fragments F1 and F2 are shown in red.

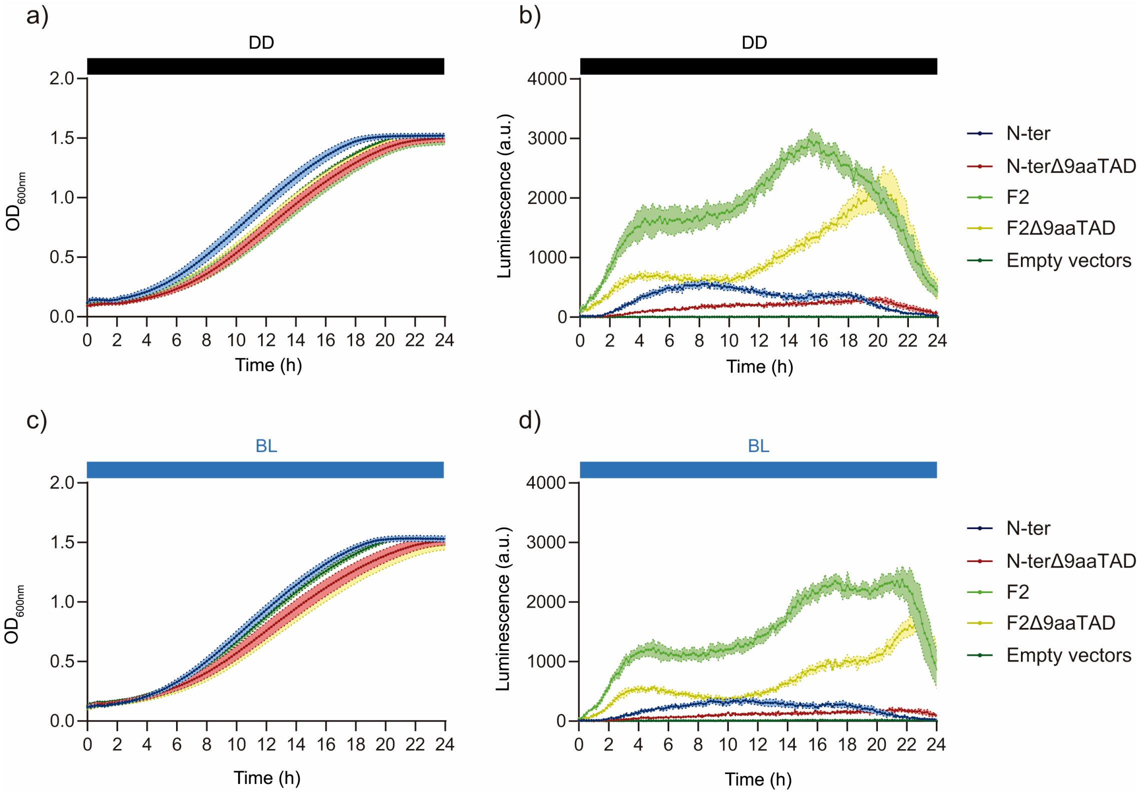

Figure S7: Raw data for chimeric transcription factors carrying a deletion in the 9aaTAD motif. The Optical Density (OD) at 600 nm (panels a and c) and the luciferase expression (panels b and d) measured as luminescence in arbitrary units (a.u.) of the yeast cultures are shown. OD and luciferase expression were measured under constant darkness (DD, panels a and b) and constant blue light (BL, panels c and d), respectively. Luciferase reporter is controlled by the *5XGAL1* synthetic promoter. In all panels, the average of six biological replicates with the standard deviation represented as a color shaded region is shown.

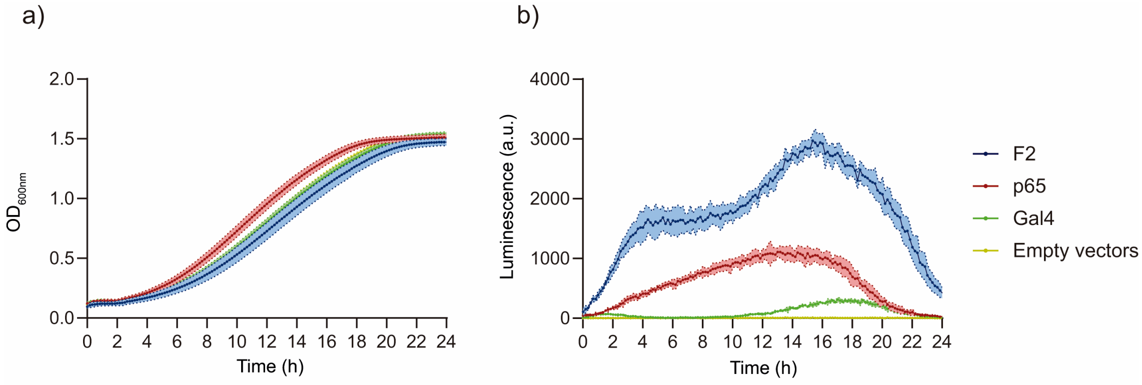

Figure S8: Raw data for chimeric transcription factors carrying Gal4 and p65 activation domains (ADs). The Optical Density (OD) at 600 nm (panels a) and the luciferase expression (panel b) measured as luminescence in arbitrary units (a.u.) of the yeast cultures are shown. Luciferase expression is controlled by the *5XGAL1* synthetic promoter under constant darkness condition. In all panels, the average of six biological replicates with the standard deviation represented as a color shaded region is shown.

Table S1: Yeast strains used and developed in this work.

Strain Name	Genotype*	Source
BY4741	*MATa his3Δ1 leu2Δ0 met15Δ0 ura3Δ0*	Euroscarf
Y685	*BY4741; plasmids 3, 4 and 18*	This work
Y686	*BY4741; plasmids 4, 18 and pRS423*	This work
Y687	*BY4741; plasmids 3, 18 and pRS425*	This work
Y688	*BY4741; plasmids 5, 6 and 18*	This work
Y689	*BY4741; plasmids 6, 18 and pRS423*	This work
Y690	*BY4741; plasmids 5, 18 and pRS425*	This work
Y691	*BY4741; plasmids 7, 8 and 18*	This work
Y692	*BY4741; plasmids 8, 18 and pRS423*	This work
Y693	*BY4741; plasmids 7, 18 and pRS425*	This work
Y409	*BY4741; plasmids 9, 18 and pRS425*	This work
Y410	*BY4741; plasmids 10, 18 and pRS425*	This work
Y411	*BY4741; plasmids 11, 18 and pRS425*	This work
Y412	*BY4741; plasmids 12, 18 and pRS425*	This work
Y679	*BY4741; plasmids 13, 18 and pRS425*	This work
Y711	*BY4741; plasmids 14, 18 and pRS425*	This work
Y680	*BY4741; plasmids 15, 18 and pRS425*	This work
Y740	*BY4741; plasmids 16, 18 and pRS425*	This work
Y710	*BY4741; plasmids 17, 18 and pRS425*	This work

* Plasmids information is shown in Table S2.

Table S2: Plasmids used and assembled in this work.

Plasmid number	Name	Genetic construct	Vector backbone	Reference
1	BcWCL1$^{PAS\Delta}$ DBD	P_{ADH1}_BcWCL1$^{PAS\Delta}$_GAL4DBD_ADH2$_{ter}$	pRS423	[25]
2	BcWCL1$^{PAS\Delta}$ AD	P_{ADH1}_BcWCL1$^{PAS\Delta}$_GAL4AD_ADH2$_{ter}$	pRS425	This work
3	sn3-BcWCL1$^{PAS\Delta}$ DBD	P_{ADH1}_sn3-BcWCL1$^{PAS\Delta}$_GAL4DBD_ADH2$_{ter}$	pRS423	This work
4	sn3-BcWCL1$^{PAS\Delta}$ AD	P_{ADH1}_sn3-BcWCL1$^{PAS\Delta}$_GAL4AD_ADH2$_{ter}$	pRS425	This work
5	sn2-BcWCL1$^{PAS\Delta}$ DBD	P_{ADH1}_sn2-BcWCL1$^{PAS\Delta}$_GAL4DBD_ADH2$_{ter}$	pRS423	This work
6	sn2-BcWCL1$^{PAS\Delta}$ AD	P_{ADH1}_sn2-BcWCL1$^{PAS\Delta}$_GAL4AD_ADH2$_{ter}$	pRS425	This work
7	sn1-BcWCL1$^{PAS\Delta}$ DBD	P_{ADH1}_sn1-BcWCL1$^{PAS\Delta}$_GAL4DBD_ADH2$_{ter}$	pRS423	This work
8	sn1-BcWCL1$^{PAS\Delta}$ AD	P_{ADH1}_sn1-BcWCL1$^{PAS\Delta}$_GAL4AD_ADH2$_{ter}$	pRS425	This work
9	Nter DBD	P_{ADH1}_Nter_GAL4DBD_ADH2$_{ter}$	pRS423	This work
10	F1 DBD	P_{ADH1}_F1_GAL4DBD_ADH2$_{ter}$	pRS423	This work
11	F2 DBD	P_{ADH1}_F2_GAL4DBD_ADH2$_{ter}$	pRS423	This work
12	F3 DBD	P_{ADH1}_F3_GAL4DBD_ADH2$_{ter}$	pRS423	This work
13	F4 DBD	P_{ADH1}_F4_GAL4DBD_ADH2$_{ter}$	pRS423	This work
14	NterΔ9aaTAD DBD	P_{ADH1}_NterΔ9aaTAD_GAL4DBD_ADH2$_{ter}$	pRS423	This work
15	F2Δ9aaTAD DBD	P_{ADH1}_F2Δ9aaTAD_GAL4DBD_ADH2$_{ter}$	pRS423	This work
16	Gal4AD DBD	P_{ADH1}_GAL4AD_GAL4DBD_ADH2$_{ter}$	pRS423	This work
17	p65 DBD	P_{ADH1}_p65_GAL4DBD_ADH2$_{ter}$	pRS423	This work
18	5XGAL1-LUC	KanMxRV_P_{5XGAL1}_Luc_CYC1$_{ter}$	pRS426	[27]

Table S3: Primers used in this work.

Name	Sequence (5′-3′)	Length (nt)	Orientation	Description
oL258	agcggataacaatttcacacaggaaacagctaggcg catgcaacttcttt	50	Fw	Recombination of *pADH1* with pRS423/pRS425
oL259	ctcgggaattaattccgctttatcggatccattcattcca ctcgttttcc	50	Rv	Recombination of BcWCL1$^{PAS\Delta}$ with GAL4AD
oL260	agtgggaaaacgagtggaatgaatggatccgataa agcggaattaattcc	50	Fw	Recombination of GAL4AD with BcWCL1$^{PAS\Delta}$
oL261	ggtaacgccagggttttcccagtcacgacggccggt agaggtgtggtcaa	50	Rv	Recombination of *ADH2ter* with pRS423/pRS425
oL451	gcgctgataagagtggcaaagagacccaatggatcc atgaagctactgtc	50	Fw	Recombination of F3/Nter BcWCL1$^{PAS\Delta}$ with GAL4DBD
oL452	ttcgatagaagacagtagcttcatggatccattgggtc tctttgccactc	50	Rv	Recombination of F3/Nter BcWCL1$^{PAS\Delta}$ with GAL4DBD
oL453	ccttcttccgagaatccaatgattcgtcgaggatccat gaagctactgtc	50	Fw	Recombination of F1 BcWCL1$^{PAS\Delta}$ with GAL4DBD
oL454	ttcgatagaagacagtagcttcatggatcctcgacga atcattggattct	50	Rv	Recombination of F1 BcWCL1$^{PAS\Delta}$ with GAL4DBD
oL455	gcatacaatcaactatctcatatacatatgcagtccttg ggtgaaatgaa	50	Fw	Recombination of *ADH1* promotor with F2/sn1-BcWCL1$^{PAS\Delta}$
oL456	gtcgttcatattcatttcacccaaggactgcatatgtat atgagatagtt	50	Rv	Recombination of *ADH1* promotor with F2/sn1-BcWCL1$^{PAS\Delta}$
oL457	accttgggagatgaaaataacgaagagatgggatcc atgaagctactgtc	50	Fw	Recombination of F2 BcWCL1$^{PAS\Delta}$ with GAL4DBD
oL458	ttcgatagaagacagtagcttcatggatcccatctcttc gttattttcat	50	Rv	Recombination of F2 BcWCL1$^{PAS\Delta}$ with GAL4DBD
oL459	gcatacaatcaactatctcatatacatatgtcatcgaga tccagtattat	50	Fw	Recombination of *ADH1* promotor with F3/sn2-BcWCL1$^{PAS\Delta}$
oL460	tgtggggcccataatactggatctcgatgacatatgta tatgagatagtt	50	Rv	Recombination of *ADH1* promotor with F3/sn2-BcWCL1$^{PAS\Delta}$
oL541	tcccctaccgtatatgggaacgaccctatggggggcc aagaccgtggtgg	50	Fw	Recombination of Nter9TAAD/F2Δ9TAAD
oL542	ggttcttatcccaccacggtcttggccccccatagggt cgttcccatata	50	Rv	Recombination of NterΔ9TAAD/F2Δ9TAAD
oL545	gcatacaatcaactatctcatatacatatggataaagc ggaattaattcc	50	Fw	Recombination of *ADH1* promotor with GAL4AD
oL546	tggaggctcgggaattaattccgctttatccatatgtat atgagatagtt	50	Rv	Recombination of *ADH1* promotor with GAL4AD
oL547	accccaccaaacccaaaaaaagagggatccatgaa gctactgtcttctat	50	Fw	Recombination of GAL4AD with GAL4DBD
oL548	tgcttgttcgatagaagacagtagcttcatggatccct cttttttgggt	50	Rv	Recombination of GAL4AD with GAL4DBD
oL549	gcatacaatcaactatctcatatacatatgcccaccca ggctggggaagg	50	Fw	Recombination of *ADH1* promotor with p65
oL550	tgacagcgttccttccccagcctgggtgggcatatgta tatgagatagtt	50	Rv	Recombination of *ADH1* promotor with p65
oL551	gccctgctgagtcagatcagctccggatccatgaagc tactgtcttctat	50	Fw	Recombination of p65 with GAL4DBD
oL552	tgcttgttcgatagaagacagtagcttcatggatccgg agctgatctgac	50	Rv	Recombination of p65 with GAL4DBD

oL658	gcatacaatcaactatctcatatacatatgaaactcatt aacataggtgc	50	Fw	Recombination of *ADH1* promotor with sn3-BcWCL1$^{PAS\Delta}$
oL659	tgttaatgagtttcatatgtatatgagatagttgattgt atgcttggtat	50	Rv	Recombination of *ADH1* promotor with sn3-BcWCL1$^{PAS\Delta}$
oL660	agcatacaatcaactatctcatatacatatgggtcgtat atcgtcgcgtag	50	Fw	Recombination of F4 with *ADH1* promotor
oL661	tttagaactacgcgacgatatacgacccatatgtatat gagatagttgat	50	Rv	Recombination of F4 with *ADH1* promotor
oL662	agtgggaaaacgagtggaatgaatggatccatgaa gctactgtcttctat	50	Fw	Recombination of F4 with Gal4DBD
oL663	ttcgatagaagacagtagcttcatggatccattcattcc actcgttttcc	50	Rv	Recombination of F4 with Gal4DBD
